# Development of a Theory-Based, Culturally Appropriate Message Library for Use in Interventions to Promote COVID-19 Vaccination Among African Americans: Formative Research

**DOI:** 10.2196/38781

**Published:** 2022-07-28

**Authors:** Jennifer Cunningham-Erves, Heather M Brandt, Maureen Sanderson, Kristin Clarkson, Omaran Lee, David Schlundt, Kemberlee Bonnet, Jamaine Davis

**Affiliations:** 1 Department of Internal Medicine Meharry Medical College Nashville, TN United States; 2 Department of Epidemiology and Cancer Control St Jude Children's Research Hospital Memphis, TN United States; 3 Department of Family Medicine Meharry Medical College Nashville, TN United States; 4 Nashville General Hospital Nashville, TN United States; 5 Department of Psychology Vanderbilt University Nashville, TN United States; 6 Department of Biochemistry and Cancer Biology Meharry Medical College Nashville, TN United States

**Keywords:** African American, Black American, Black, minority, ethnic, culturally sensitive, cultural sensitivity, inclusive, vulnerable, COVID-19, vaccination, vaccine, health promotion, campaign, messaging, culturally appropriate, theory, adults, children, disparity, health belief model, community engagement, public engagement, public awareness, community-based, health information, health communication, health intervention, vulnerable population, community health, patient education

## Abstract

**Background:**

Disparities in COVID-19 incidence, hospitalization, and mortality rates among African Americans suggest the need for targeted interventions. Use of targeted, theory-driven messages in behavioral and communication interventions could empower African Americans to engage in behaviors that prevent COVID-19.

**Objective:**

To address this need, we performed a formative study that aimed to develop and design a culturally appropriate, theory-based library of messages targeting concerns around COVID-19 vaccines that could be used in behavioral and communication interventions for African Americans.

**Methods:**

Message development occurred between January 2021 and February 2022. Initial messages were designed by a multidisciplinary team of researchers, community leaders, and community members. Kreuter’s 5 strategies (ie, linguistic, peripheral, evidential, sociocultural, and constituent-involving strategies) were used to achieve cultural appropriateness. After forming a community-academic partnership, message development occurred in 4 phases: (1) adaptation of a message library using the literature, (2) review by 6 clinical and research experts for content validation, (3) input and review by a 6-member community advisory panel (CAP), and (4) message pretesting with African Americans via semistructured interviews in a qualitative study.

**Results:**

Themes from the semistructured interviews among 30 African Americans were as follows: (1) community reactions to the messages, (2) community questions and information needs, (3) suggestions for additional content, and (4) suggestions to improve comprehension, relevance, and trustworthiness. Feedback from the CAP, community members, and scientific experts was used by members of the community-academic partnership to iteratively update message content to maximize cultural appropriateness. The final message library had 18 message subsets for adults and 17 message subsets for parents and caregivers of children. These subsets were placed into 3 categories: (1) vaccine development, (2) vaccine safety, and (3) vaccine effectiveness.

**Conclusions:**

We used a 4-phase, systematic process using multiple community engagement approaches to create messages for African Americans to support interventions to improve COVID-19 vaccination rates among adults and children. The newly developed messages were deemed to be culturally appropriate according to experts and members of the African American community. Future research should evaluate the impact of these messages on COVID-19 vaccination rates among African Americans.

## Introduction

### Background

Since December 2020, 3 vaccines have been approved in the United States to prevent severe disease and death caused by COVID-19. The 2-dose vaccinations developed by *Pfizer* and *Moderna* are approved for use in individuals aged 18 years and older and under emergency use authorization (EAU) for children aged 6 months to 17 years [1]. The 1-dose *Johnson & Johnson* vaccination is under EAU for adults aged 18 years and older [1]. Although highly effective [2], vaccination rates remain suboptimal, especially among populations who could benefit most. For example, African Americans comprise 12.4% of the US population but only 10.1% of those who have initiated the series and 10.3% of those who have completed the series as of March 10, 2022 [3]. Despite African Americans being almost 2 times more likely than White Americans to die from COVID-19 [4], vaccine hesitancy remains a major hindrance to reduced vaccine uptake among African Americans [5-9].

Emerging studies demonstrate that vaccine hesitancy is deeply rooted in several overlapping areas: (1) mistrust in health care, government, and research [10-13]; (2) structural racism [14]; and (3) lack of understanding of science related to vaccine-specific issues (eg, efficacy, safety, speed of development) [13,15]. Lack of information, misinformation, and disinformation further drive vaccine hesitancy [10], with social or mass media as the primary source [15]. Because effective communication is necessary to help African Americans make informed decisions about COVID-19 vaccines [16], studies have begun to explore the communication strategies necessary to increase COVID-19 vaccination [17-19]. Trusted messengers are key to COVID-19 information being well received and used [20].

The specific messages related to COVID-19 vaccination are as important as the messenger. Information sources have been developed and disseminated widely to educate communities on messages to use to educate communities on COVID-19 vaccination to increase uptake [21,22]. A few emerging studies have tested messages, including persuasive messaging [18], video-based messages [23], and behavioral nudges [24], on vaccination intention or uptake. African Americans suggest the need for messages that are accurate, targeted, culturally appropriate, and community based [25,26]. However, perceptions of the messages remain unknown, and none (to the best of our knowledge) have actively engaged the African American community to develop or refine the messages on COVID-19 vaccination to ensure cultural appropriateness. Such engagement is key because African Americans’ values and decision-making about the COVID-19 vaccine are strongly shaped by culture [10,12,13,27], and targeting will maximize “fit” of information to an individual’s unique characteristics [28,29].

### Conceptual Framework

Message development was guided by 2 psychosocial decision-making models: (1) theory of reasoned action (TRA) [30] and (2) the Health Belief Model (HBM) [31]. These health behavior theoretical models are commonly used to understand vaccination decision-making. The TRA predicts that behavioral intentions to vaccinate against COVID-19 are based on attitudes and subjective norms. The HBM predicts that the likelihood of vaccinating against COVID-19 is based on perceived susceptibility of the individual to SARS-CoV-2, perceived severity of COVID-19, and whether perceived benefits of vaccination outweigh perceived barriers.

Kreuter et al [32] proposed 5 strategies to achieve cultural appropriateness that were used to guide message development. *Peripheral strategies* increase communication appeal through the title, fonts, colors, and images. *Evidential strategies* provide data on impact of a health issue in a certain group. *Sociocultural strategies* address health issues from the social and cultural values of a group. *Linguistic strategies* fit the program to the native language of a certain group. Lastly, *constituent-involving strategies* ensure community members’ inclusion in program planning. Developing culturally appropriate, theory-based messages that can be used in communication and behavioral interventions may address concerns about COVID-19 vaccination among African Americans. Because social marketing campaigns have been effective in changing knowledge, attitudes, intentions, and behavior at the community level [33-35], they can disseminate theory-based, culturally appropriate messages and potentially increase COVID-19 vaccination acceptability and uptake. Social marketing is “the application of proven concepts and techniques drawn from the commercial sector to promote changes in diverse socially important behaviors such as drug use, sexual behavior…This marketing approach has an immense potential to affect major social problems if we can only learn how to harness its power” [34].

### Study Objectives

We describe the development of a theory-based, culturally appropriate library of motivational messages for a social marketing campaign to promote COVID-19 vaccination among African Americans who are vaccine hesitant. Message development occurred in 4 phases: (1) adaptation of a message library based on the literature, (2) review by clinical and research experts for content validation, (3) input and review by a community advisory panel (CAP), and (4) message pretesting via a qualitative study with African American community members to evaluate the accuracy, relevance, and persuasiveness of the messages. The long-term goal is for these messages to be used within interventions aimed at increasing COVID-19 vaccination among African Americans.

## Methods

### Study Design

**Figure 1:**
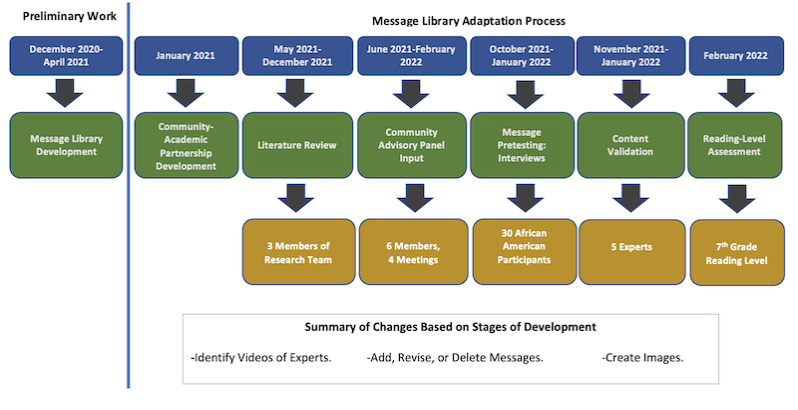
Message library development process.

We conducted a formative study to design and develop a theory-based, culturally appropriate message library that could be used in behavioral and communication interventions to increase COVID-19 vaccine uptake among African Americans. In the existing COVID-19 library, 1 message subset for adults and 1 for parents and caregivers of adolescents was developed and iteratively adapted by coauthors JCE (a behavioral scientist with a background in biology and vaccine hesitancy) and JD (a basic scientist with a background in infectious disease) on the basis of emerging literature and feedback from over 30 educational sessions provided to communities on COVID-19 and the vaccine. A community-academic partnership was formed to iteratively adapt the message library over a 1-year period to ensure that it was theory based, culturally appropriate, and up to date. Our adaptation process occurred through 6 phases, with community input at each phase. See Figure 1 for a depiction of the 1-year process to yield the final message library of adult and parent message sets.

### Development of a Community-Academic Partnership, January 2021

A community-academic partnership was developed between 2 academic partners, Meharry Medical College and St Jude Children’s Research Hospital, and 1 community partner, the Congregational Health and Education Network (CHEN) in 2021. This partnership yielded an interdisciplinary team of experts in basic science, epidemiology, behavioral science, communication, and community engagement. The purpose of this partnership was (1) to develop messages and products for a social marketing campaign using community engagement principles and (2) to implement and evaluate the social marketing campaign. CHEN, a 501c(3), is a collaboration between Nashville General Hospital at Meharry Medical College and other academic institutions, along with faith-based organizations serving African American and Latino congregants [36]. A focus for CHEN during the COVID-19 pandemic was to improve vaccine health literacy and uptake to reduce health inequities in vaccination and related outcomes. The community partner (CHEN) ensured that the messaging represented the top vaccination concerns and was culturally appropriate. The academic partners offered guidance, when needed, to ensure that the partnership was equitable, and bidirectional engagement occurred in all research phases. They also ensured scientific accuracy of messages.

### Adaptation of the Existing Message Library, May-January 2022

Members of the research team conducted a literature search to identify reasons for COVID-19 vaccine hesitancy and acceptance, along with potential strategies to improve coverage among African Americans. See Multimedia Appendix 1 for the methods and results of the search. This search was conducted from May 2021 until January 2022 to ensure that the library had the latest updates before the launch of the social marketing campaign. Using the literature, the TRA [30] and the HBM [31], and the experiences and skills of the research team, we adapted the existing message library to ensure accuracy and relevance.

### Input from the Community Advisory Panel, June 2021-February 2022

An 8-member CAP was formed to provide feedback on concerns about the vaccine and potential strategies to increase vaccination. This panel meets quarterly and comprises African Americans in the Nashville, Tennessee, metropolitan statistical area. Organizations represented included the Matthew Walker Comprehensive Health Center, Health Leads, the National Association for the Advancement of Colored People, the Community Partners’ Network, and the Nashville Health Disparities Coalition. Additional members included a young adult, a physician, and a parent. A subgroup of the CAP (n=4, 50%) reviewed the content and provided feedback on the messages. The feedback from the meetings and content review from the subgroup were used to iteratively develop the messages and images for the library.

### Message Pretesting With African Americans, October 2021-January 2022

#### Study Design

We conducted a phenomenological, qualitative study [37] to create messages that could be used to assist in decision-making on COVID-19 vaccination among African Americans. Specifically, we conducted semistructured interviews (1) to identify reasons participants decided to receive or decline the vaccine for their self or child and (2) to gain feedback on messaging relevance, acceptability, and comprehensibility. This protocol was guided by the HBM [31], the TRA [30], and the community partner (CHEN) and CAP input. Social marketing campaign content (ie, draft messages, images/graphics) was iteratively revised using the data.

#### Sampling and Recruitment

We recruited a purposive sample of African Americans in the southeastern United States who met the following eligibility criteria: (1) adults aged 18 years or older and vaccinated or unvaccinated and (2) parents or caregivers of children aged 5-18 years and vaccinated or unvaccinated. Our community partner (CHEN) and the CAP members recruited participants via email, telephone, or word-of-mouth by using their social network and existing databases. ResearchMatch (RM), an online research recruitment tool, was also used for recruitment [38].

#### Data Collection

Interested participants completed a screener. If they qualified, then they completed informed consent procedures and a brief 12-item survey on barriers to COVID-19 vaccination, with response options on a 5-point Likert-type agreement scale. The screener, consent, and demographic survey were completed via Research Electronic Data Capture (REDCap; Vanderbilt University) [39], a secure web-based data collection application. Then, participants were emailed a copy of the adult and parent message sets a minimum of 3 days before the interview.

Participant interviews were conducted by a trained interviewer and lasted 45-60 minutes. Open-ended scripted questions were asked using an interview guide. Questions included (1) attitudes and beliefs about COVID-19 and vaccination, (2) facilitators and barriers to COVID-19 vaccination, (3) message library content and images, and (4) dissemination strategies. For the message development section of interviews, specific questions included the following:

- What is your overall view and purpose of the message?
- Which parts of this message did you not understand or were not clear?
- What should be added to the message?
- What message should be removed or changed?

Follow-up questions were asked for clarification and to facilitate in-depth discussion. Participants were paid a US $50 gift card. Interviews were recorded, transcribed verbatim, and de-identified for data analysis.

#### Data Analyses

Qualitative data coding and analysis was managed by the Vanderbilt University Qualitative Research Core, led by a PhD-level psychologist. Data coding and analysis were conducted following Consolidated Criteria for Reporting Qualitative Studies (COREQ) guidelines, an evidence-based qualitative methodology [40]. A hierarchical coding system was developed and refined using the interview guide and a preliminary review of the transcripts. Experienced qualitative coders first established reliability in using the coding system on 2 transcripts, reconciling any discrepancies, and then independently coded the remaining transcripts. We used an iterative inductive/deductive approach to qualitative data analysis [41,42]. Inductively, we sorted the quotations by coding category to identify higher-order themes and relationships between themes. Deductively, we were guided by the HBM and the TRA. The transcripts, quotations, and codes were managed using Microsoft Excel 2016 and IBM SPSS Statistics version 28.0. Survey data were also analyzed using SPSS Statistics version 28.0. Descriptive analysis (eg, means, frequencies) and bivariate analysis (eg, chi-square tests, Fisher exact tests) were performed to describe patterns in the data.

#### Message Library Revision Process

Using the original message library, the research team iteratively modified the message sets and added visuals and videos to “match” the messages to increase comprehensibility and appropriateness. A subgroup of the research team met regularly to discuss interview findings and the message library, and subsequent changes were made. Then, all members of the partnership met to discuss the changes and identify additional needed modifications. Each new iteration of the messages and visuals was developed using peripheral, evidential, linguistic, and sociocultural strategies [32]. A final meeting was held to ensure that all feedback was incorporated into the message library and was culturally appropriate.

### Content Validation of Messages by Experts, November 2021-January 2022

Five experts were identified to review the content for accuracy and relevance. These reviews were conducted strategically due to the ever-changing nature of COVID-19 pandemic updates. Specifically, 2 experts reviewed the content prior to, 1 during, and 3 after community review. Selection criteria for these content reviewers were experience in SARS-CoV-2/COVID-19 research, including vaccination, expertise in the use of psychosocial theory, and willingness and ability to review the items.

Adapted from Lawshe [43], we used qualitative and quantitative methods in a 2-phase content review process. Specifically, each message subset was quantitatively evaluated for relevance by using a 3-point Likert scale: “essential,” “useful but not essential,” and “not necessary.” Then, experts reviewed the messages for perceived accuracy and clarity by providing written comments and edits for improvement. Using these methods, we iteratively refined the messages.

### Ethical Considerations

This research was approved by Meharry Medical College’s Institutional Review Board (Protocol 21-03-1076). All participants provided oral informed consent.

## Results

### Community Advisory Panel Feedback

The CAP members’ feedback evolved throughout the message library development process. At the beginning of each meeting, the researchers gained insight into the community’s thinking about the COVID-19 vaccine to determine trends in hesitancy. Top concerns included vaccine safety, the speed of vaccine development, mistrust in research and health care, politicization of the vaccine, and conspiracy theories (eg, tracking chip in the vaccine). We then asked about the presentation of messaging. Members suggested that messages should be concise yet comprehensible across reading levels. Members further indicated the need to discuss immediate and long-term benefits and the risks of vaccination so that the community can make an informed decision. A few members further suggested the need to use numbers and images to explain these concerns more clearly.

Select members of the CAP were asked to conduct a detailed review of the messages and images to identify ways to make the content more relatable and comprehensible. Some even provided preferred sources of content (eg, NPR (National Public Radio), which is media organization that seeks to create a more informed public via air, online, or in-person) to help develop the material. Collectively, we used the feedback from the quarterly advisory panel meetings and subgroup review of messages to update the library.

### Semistructured Interviews

#### Sociodemographics

Most participants were female and had a college degree or higher. More than half had a household income less than US $80,000. The mean age was 38.6 years (SD 9.49 years). See Table 1 for sociodemographics by subgroup: vaccinated adult, unvaccinated adult, adult with unvaccinated child, and adult with vaccinated child.

**Table 1:** Sociodemographics of African American interview participants (N=30).

Characteristic		Parent^a^ with vaccinated child (N=7)	Parent with unvaccinated child (N=7)	Adult, vaccinated (N=9)	Adult, unvaccinated (N=7)
Age (years), mean (SD)		42.4 (6.1)	37.4 (6.2)	36.1 (12.5)	39.1 (11.1)
**Gender, n (%)**					
	Male	2 (29)	1 (14)	2 (22)	1 (14)
	Female	5 (71)	6 (86)	7 (78)	6 (86)
**Education, n (%)**					
	Some college or lower	2 (29)	2 (29)	2 (22)	1 (14)
	College degree or higher	5 (71)	5 (71)	7 (78)	6 (86)
**Household income (US $), n (%)**					
	≤40,000	2 (29)	2 (29)	2 (22)	3 (42)
	40,001-80,000	2 (29)	0	2 (22)	2 (29)
	>80,000	3 (42)	4 (57)	3 (34)	0
	Not available	0	1 (14)	2 (22)	2 (29)

^a^Parents had children aged 5-18 years.

#### Summary of Findings

Using the inductive-deductive approach, we identified 4 primary themes specific to the development of messages related to COVID-19 and the vaccines in the qualitative study. Messages were referenced in the text by the theme and message number (eg, 1.01 is the first quotation related to theme 1). See Multimedia Appendix 2 for quotations related to each theme.

#### Theme 1: Community Reactions to the Messages

Overall, the community members found that the messages were “very helpful” and had a “community feel.” Specifically, the messages were inclusive and comprehensive and had a good balance between science and simple language. The messages were viewed as “good,” “persuasive” (quotation 1.02), and “educational” (quotations 1.01-1.02). Most participants stated that the reading level was good, and suggested a few edits to specific messages (eg, whether the messenger RNA [mRNA] vaccine changes your DNA). Although there were mixed reviews, most perceived the length and number of messages in each set to be appropriate.

For message presentation, many perceived that there were good analogies and comparisons to increase comprehension (quotation 1.03). If applicable to the message set, participants liked the balance of benefits and risks of vaccines. Lastly, a few participants cited the messages as relatable, trustworthy, and credible. Collectively, participants perceived that the information helped guide decision-making and did not simply “tell you” to take the vaccine.

#### Theme 2: Questions and Information Needs

Some participants had questions after reviewing the messages. A participant wanted to know where specific evidence for the general numbers on some websites (eg, the Centers for Disease Control and Prevention [44]) could be found. Other questions were related to long-term effects of the vaccine, “why we even need the vaccine,” mixing vaccines and boosters, or the number of boosters after the first dose (quotation 2.01). Others asked about the relationship of COVID-19 vaccines and fertility. Lastly, participants asked how existing conditions (eg, diabetes, asthma) were related to the severity of COVID-19.

#### Theme 3: Suggestions for Additional Content

Most participants had suggestions for additional content or context for specific message sets. A suggestion for overall messages was to add a statement that science evolves as more data are collected to communicate new findings, which are constantly being added to increase our knowledge of COVID-19. For vaccine safety, additional information was requested on mRNA, along with the 30-year history of studies of mRNA and its use in vaccines (quotation 3.01). Participants asked for more information on the clinical trial process and better justification for boosters and their side effects. As it relates to vaccines and infertility, a suggestion was made to provide recommendations from gynecology professionals and experts and information about long-term effects of COVID-19 vaccines on infertility. Lastly, a few participants wanted to know whether the vaccines can lead to sexual dysfunction (quotation 3.02).

Distrusting in the government and pharmaceutical companies, participants wanted more details about the COVID vaccines (ie, development, testing, and ingredients; quotation 3.03). For vaccine effectiveness, participants asked about healthy people getting the virus and how outcomes of the vaccinated compare to those of the unvaccinated (quotation 3.04). They also asked why natural immunity is not better than vaccine-induced immunity. Participants wanted more information on variants (eg, Omicron), their severity, and how variants affect vaccine effectiveness.

For children specifically, participants asked for data on successes and challenges during development (quotation 3.05), along with updates on vaccine safety. Participants also suggested reinforcing other preventive behaviors, such as sanitizing, healthy eating, and physical distancing (quotation 3.06). Some further wanted to know alternative ways to boost their immune system without taking a vaccine or booster (quotations 3.07 and 3.08). A few suggested the need to encourage conversations with doctors, especially for those with underlying medical conditions, prior to getting the vaccine.

#### Theme 4: Suggestions to Increase Comprehension, Relevance, and Trustworthiness

A few suggestions were made to enhance comprehension. One suggestion was to provide definitions for specific terms (eg, high risk; quotation 4.01). Providing an easier-to-understand presentation of statistics was commonly mentioned. For example, participants further suggested the “need for statistics or more data” to help understand the vaccine development process. Participants emphasized the importance of clarifying the magnitude of potential side effects. Visuals were suggested to improve understandability of messages. Participants also suggested the use of specific terms such as “vaccination” and not “shot.” Lastly, 1 (3%) participant suggested having information available in other languages (eg, Arabic).

A few participants indicated the need for messages to be tied to things people already understand, such as flu or smallpox vaccines, to increase relevance. Other suggestions were to use videos and images to reflect content. Some participants also wanted videos of personal testimonies of individuals who were undecided about the vaccine and their decision-making process to get vaccinated (quotation 4.02). Notably, testimonials were also perceived by others to be too contrived (quotation 4.03). To increase trustworthiness, participants suggested providing proof that doctors or health professionals (quotation 4.04) supported this work, along with the addition of informational sources, especially links to studies that provide supportive evidence. Lastly, participants encouraged honesty and transparency in information related to the COVID-19 vaccines.

### Content Validation

In total, 6 reviewers validated the content. Of these 6, 2 (33%) were White Americans, and 4 (67%) were African Americans; 4 (67%) were female, and 2 (33%) were male. In addition, 1 (17%) reviewer provided feedback for only adult concerns. Reviewers’ areas of expertise included vaccine development, immunology, vaccine-preventable disease and immunizations, and nurse safety. Messages were iteratively updated based on feedback of experts. Most message subsets were classified as essential or useful but not essential. Content edits and additions to the message library reflected the updates on the coronavirus and the vaccine and strategies to ensure comprehensibility and accuracy. Reordering, rephrasing, and adding (eg, analogies) of content were conducted to increase clarity. Because updates are ongoing for COVID-19, content was deemed evidential once primary concepts (eg, process of vaccine development or purpose of boosters) were validated, with the intent to continue to update the library with expert review.

### Reading Grade Level Assessment

To finalize the library messages, a readability assessment was conducted. We used 3 primary reading grade level assessment tools and an online consensus tool to assess the reading grade level of the adult and parent message sets. First, the Flesch Reading Ease Score [45] was calculated in Microsoft Word. Higher scores indicate easier readability by the user. Second, the Flesch-Kincaid Grade Level [46] was calculated in Microsoft Word to determine a US school grade level. The Flesch Reading Ease and Flesch-Kincaid Grade Levels are calculated by considering the average sentence length (total number of words divided by total number of sentences) and the average syllables per word (total number of syllables divided by the total number of words) using different underlying formulas. Third, the Simple Measure of Gobbledygook (SMOG) index was hand-scored, in addition to being automatically calculated to determine a US [47] school grade level. The SMOG index is calculated using the number of words with multiple syllables in three 10-sentence samples at the beginning, middle, and end of the text. These 3 readability assessment tools all use word difficulty and sentence length as the main factors in determining how easy or how hard the material is to read. Finally, an online readability calculator [48] was used to determine readability consensus. The online calculator applied 7 commonly used readability formulas to provide a consensus rating. See Table 2 for readability assessment results.

The readability assessment revealed acceptable reading grade level scores for all readability formulas applied. The usual recommended reading grade level is fifth-sixth grade to optimize comprehension, according to the American Medical Association and the United States Department of Health and Human Services. However, in combination with the iterative development process and ongoing review by the community partner (CHEN), the CAP, and experts, the messages reflected use of plain language when polysyllabic or complex words were unavoidable.

**Table 2:** Reading grade level results.

Readability assessment tool	Adult library	Parent library
Flesch Reading Ease Score	62.0 (standard/average)	62.5 (standard/average)
Flesch-Kincaid Grade Level	8.4 (8th grade)	8.3 (8th grade)
SMOG^a^ index	7.7 (8th grade)	7.6 (8th grade)
Readability consensus^b^	Grade level: 8 Reading level: standard/average Age of reader: 12-14 years (7th-8th grade)	Grade level: 8 Reading level: standard/average Age of reader: 12-14 years (7th-8th grade)

^a^SMOG: Simple Measure of Gobbledygook.

^b^Readability consensus was based on the application of 7 readability formulas using an online calculator available [44].

### Message Set Finalization

After completion of the readability assessment, members of the research team conducted a final review to ensure comprehensibility and accuracy. Minor edits were made. See Table 3 for an example of a concern, along with an example of a message for the concern after the adaptation process.

**Table 3:** Example of a message for tailoring a variable postiterative development process.

Vaccine concern			Example message
**Category 1: vaccine development**			
	“Human protections in research” [adult and parent]	Many people wonder about taking part in research and if they will be protected. We know there have been past research studies that were not done right [provides examples of historical research abuses]. To begin to address this issue, we give a few examples to show every person is protected when they take part in research and how the community could benefit after the research study is done [provides examples of human protections in research].	
	“Who is at the table?” [Adult and parent]	Many people wonder if people of all racial backgrounds were involved when the vaccines were developed. Individuals from all races were at the table to help guide the process [provides examples of scientists of all racial/ethnic backgrounds and their role in the development process].	
**Category 2: vaccine safety**			
	“mRNA^a^ and DNA” [adult]	We all have mRNA in every cell in our bodies. mRNA is known as messenger RNA. It is the “recipe” that tells the cells in our body to make certain proteins. The mRNA protein in the Pfizer and Moderna COVID-19 vaccines shows up, teaches the immune system how to develop antibodies against SARS-CoV-2 (the virus that causes COVID-19), and then quickly dissolves. mRNA never enters the nucleus of the cell where your DNA is kept. Your body learns how to protect itself against future SARS-CoV-2 infection without ever having to risk getting the virus or the serious outcomes of getting sick with COVID-19.	
	“Infertility” [adult]	The antibody to the spike protein does not make a woman infertile or unable to get pregnant. There was a false claim that there were similarities between the SARS-CoV-2 spike protein and the surface of a protein on placental cells. Placental cells are needed for a successful pregnancy. SARS-CoV-2 spike protein and the placental cells are not the same. This means the vaccine will not cause the immune system to make antibodies against the placental protein.	
	“Your child’s heart” [parent]	Many parents ask about the COVID-19 vaccine and how it can affect the hearts of children. In the United States, there has been an increase in myocarditis and pericarditis cases after getting the mRNA COVID-19 vaccine. Myocarditis is mild inflammation of the heart. Pericarditis is mild inflammation of the sack around the heart. For children under 16 years of age, myocarditis risk is 37 times higher for children with COVID-19 than the children without COVID-19. So, myocarditis does not happen often. The American Academy of Pediatrics says children and teens should get the COVID-19 vaccines.	
	“My child has underlying medical conditions.” [Parent]	The Pfizer vaccine can be given to children 5 [years] and older with underlying medical conditions like diabetes or autoimmune diseases. It cannot cause COVID-19, even in those with weak immune systems. Children with underlying medical conditions took part in the clinical trials and serious reactions to the vaccine [were] rare. However, children with underlying medical conditions are more apt to have problems from COVID-19.	
**Category 3: vaccine effectiveness**			
	“Boosters. Why?” [Adult]	All routine [vaccines] require booster doses to have full protection [adds examples]. So the COVID-19 vaccine is not any different. Booster shots are given to jumpstart the body’s immune system to produce more antibodies against the original SARS-CoV-2 (the virus that causes COVID-19) and help protect against new variants. Because antibody levels decrease over time, boosters are needed to keep us protected.	
	“Variants and the vaccine” [adult]	As SARS-CoV-2 (the virus that causes COVID-19) continues to infect people, it is more likely to mutate. This means that the virus makes a new version of itself also known as a mutation. It is common for this to happen. Mutations affect how viruses work, like to help the virus better attach to our cells or lower the virus’s ability to attach to our cells. So it is important for people to complete COVID-19 vaccination. More mutations and new variants may lower or stop the protection provided by the vaccines.	
	“Natural immunity or vaccine immunity”	Natural immunity happens when your child’s body gets infected with the SARS-CoV-2 virus, the virus that causes COVID-19. While your child’s body will make antibodies against the virus, the danger is in your child getting very sick and maybe even dying. Immunity from getting a vaccine is very similar to immunity developed through natural infection but does not carry the increased risk of your child getting very sick or even death. Natural immunity provides less protection over time than the immunity gained by COVID-19. While people can gain immunity after getting the virus, studies show that more than one third of COVID-19 infections results in low levels of protective antibodies.	
	“Too many vaccines” [parent]	We all got different vaccines when we were babies, adolescents, and event adults [gives an example of multiple vaccines being given]. These vaccines are routinely given at the same time without serious side effects. So, getting more than one vaccine is something we have been doing since birth.	

^a^mRNA: messenger RNA.

### Final Message Library

The final message library had 2 message sets, 1 for adults and 1 for parents. There were 18 message subsets for adults and 17 message subsets for parents. These subsets were placed into 3 categories: vaccine development, vaccine safety, and vaccine effectiveness. Each message subset begins with expressing empathy toward the individuals’ concern. Then the facts are provided around each concern, positive or negative. Lastly, the message subset ends with a positive statement related to COVID-19 vaccination that addresses concerns. All message subsets were reviewed by community leaders and members (constituent-involving strategy). We briefly describe each subset for each group next. Each message subset was presented using 3 modes: content, image, and video. See Table 4 to identify each concern, along with key message attributes and the associated strategy to achieve cultural appropriateness.

**Table 4:** Vaccine concern, key message attributes, types of visuals, and type of cultural targeting strategy.

Vaccine concern^a^			Key message attributes		Types of visuals		Cultural targeting strategy
**Vaccine development**							
	Human protections in research	Acknowledge historical research abuses (eg, U.S. Public Health Service Syphilis Study, Henrietta Lacks). Provide examples of protections provided in research. Discuss how the community can be involved in the research process (eg, co–primary investigator, community advisory board, consultant).		Not applicable		Sociocultural, evidential	
	Who is at the table?	Discuss researchers (ie, current job, expertise) across all backgrounds and their role in the development process. List their current jobs.		Researchers of diverse backgrounds (visual) Researcher describing role in vaccine development (video)		Sociocultural, evidential, peripheral	
	Too new and too quick	Define mRNA^b^ and its role in the body. Discuss the mRNA vaccine history and how it works in the body. Define what mRNA does not do (change DNA). Compare mRNA vaccine development to existing vaccine development processes. Define the EUA^c^. Discuss the number of vaccines given to date, adverse events, and how to identify those events. Confirm that being unvaccinated places one at higher risk of death compared to those who received the vaccine.		Timeline of COVID-19 vaccine development (visual and video)		Evidential, peripheral	
	How research works	Define research. Define clinical trials and their phases. Define types of researchers. Define sites of research and who can participate. Discuss what happens after research.		Demonstration of phases in the clinical process and steps within each phase (visual)		Evidential, peripheral	
**Vaccine safety**							
	mRNA and DNA	State years of mRNA existence and mRNA’s role in the body. Explain the process of mRNA technology. Identify vaccines that use mRNA technology. Emphasize benefits of vaccination over natural immunity.		Demonstration of the mRNA technology process (visual and video)		Evidential, peripheral, linguistic	
	Infertility	Demonstrate how the proteins needed for pregnancy and needed to make the spike protein are not the same. Emphasize that women are able to conceive, have a healthy pregnancy and baby, and breastfeed after vaccination. State that babies receive antibodies from vaccinated mothers. Highlight that COVID-19 may impact fertility in men. Highlight cons of nonvaccination in pregnant women (eg, increased risk of stillbirth, newborn deaths, hospitalization).		Explanation of pregnant women getting the vaccine or women conceiving getting vaccinated (video)		Evidential, linguistic	
	Underlying medical conditions	State the reason to vaccinate with an underlying condition. State that the vaccine will not give an individual COVID-19. Discuss the vaccine schedule for those immunocompromised. Discuss the severity in COVID-19 if not vaccinated. State to consult with a doctor in getting the vaccine.		Explanation of why those with underlying medical conditions need the vaccine (video)		Evidential, linguistic	
	Your heart	Define myocarditis and pericarditis. Compare the rate of heart problems in those who get vaccinated compared to those who get COVID-19. Demonstrate the symptoms and treatment of heart disorders. Emphasize the recommendation by infectious disease experts and the American Academy of Pediatrics for children.		Explanation of myocarditis and vaccination (video)		Evidential, linguistic	
	GBS^d^ (adult only)	Define GBS. Discuss the signs of GBS. Discuss the number of cases to date after vaccination. Emphasize that it is rare.		Not applicable		Evidential, linguistic	
	Blood clots (adult only)	Identify the number of cases with the Johnson & Johnson vaccine. Discuss why the Food and Drug Administration (FDA) halted the clinical trial to determine whether risks of blood clots outweigh the benefits of the vaccine. State recommendation of Moderna and Pfizer vaccines over the Johnson & Johnson vaccine. Discuss the symptoms of blood clots. State the blood clot risk for those vaccinated and unvaccinated.		Symptoms of blood clots vs symptoms of COVID-19 (visual)		Peripheral, evidential, linguistic	
	Side effects	Emphasize the number of years for COVID-19 research. Discuss the number of lives and hospitalizations prevented with vaccines. Identify the risk of allergic reactions and short-term side effects. Discuss that side effects are short-lived and everyone reacts differently. State that routine vaccinations show no long-term side effects.		Side effects of vaccination compared to natural infection through SARS-CoV-2 (visual and video)		Peripheral, evidential, linguistic	
	Too young (parent only)	Emphasize the impact of COVID-19 on children. Provide recommendations for COVID-19 vaccination by age. Emphasize that vaccination protects them and others. State that the long-term effects of COVID-19 in children are unknown, but long COVID is seen in many.		Statistics of current COVID-19 cases in children and increases in COVID-19 cases, hospitalizations, and deaths in children overtime (visual)		Evidential, peripheral, linguistic	
**Vaccine effectiveness**							
	Unsure if it works	Define effectiveness and how to obtain it (ie, fully vaccinated). Demonstrate risks if not vaccinated.		Comparison of risk of hospitalization and death of those vaccinated vs not vaccinated (visual)		Peripheral, evidential, linguistic	
	Variants	Define “breakthrough case.” Discuss mutations and how new variants are created. Discuss the impact of emerging variants on vaccines and health. Emphasize the impact of virus on short- and long-term health.		Not applicable		Evidential, linguistic	
	Natural immunity	Define natural immunity versus vaccine-induced immunity. Emphasize vaccine-induced immunity being much safer than natural immunity. Discuss the “gamble” in natural immunity over vaccine-induced immunity. Discuss the benefits of vaccination despite having COVID-19.		Comparison of the health risks of those with natural immunity and those vaccinated (visual and video)		Peripheral, evidential, linguistic	
	Too many vaccines	Discuss the lack of danger of multiple vaccines at a time. Remind people of receiving many vaccines at once as a baby and preteen. Compare the number of proteins in the vaccine to the number of proteins if exposed to SARS-CoV-2.		Not applicable		Evidential, linguistic	
	Boosters. Why?	Define boosters and why they are needed. Discuss booster recommendations. Emphasize discussing getting a booster with a provider.		Stating the vaccine dose and booster schedule of each vaccine (image) Defining a booster and why we need it (video)		Evidential, peripheral, linguistic	
	Is it even needed?	Discuss transmission routes and rates by variant. Compare COVID-19 hospitalization, long COVID, and death rates among those vaccinated and unvaccinated. Discuss the susceptibility and severity of COVID-19 and the importance of vaccination.		Tracker of COVID-19 rates and deaths (United States and Tennessee)		Evidential, peripheral, linguistic	

^a^All vaccine concerns were vetted by community leaders and members (ie, constituent-involving strategy) and edited to be comprehendible (ie, linguistics).

^b^mRNA: messenger RNA.

^c^EAU: emergency use authorization.

^d^GBS: Guillain-Barré syndrome.

#### Vaccine Development

These message subsets target individuals who have concerns about the COVID-19 vaccines and the development process. The goal is to positively influence attitudes toward researchers and the process. There are 4 message sets in this category:

- Human protections in research/child protections in research
- Who is at the table?
- Too new and too quick
- How research works

#### Vaccine Safety

These message subsets target individuals who have concerns about the safety of the COVID-19 vaccines. The goal is to demonstrate that the benefits of COVID-19 vaccination outweigh the harms of COVID-19 vaccination. There are 8 message sets in this category:

- mRNA and DNA
- Infertility/youth infertility
- Underlying medical conditions
- Your heart/your child’s heart
- Guillain-Barré syndrome (GBS; adults only)
- Blood clots (adults only)
- Side effects
- Child is too young (parents only)

#### Vaccine Effectiveness

These message subsets seek to demonstrate that the risk of SARS-CoV-2 and the severity of COVID-19 (ie, long-haul COVID-19, hospitalization, and death) are far greater when not vaccinated against COVID-19. These sets further demonstrate that the vaccine is effective and how variants may affect effectiveness. We also discussed the dosing schedule and role of boosters. There are 7 message sets in this category:

- Unsure if it works
- Variants and the vaccine
- Natural immunity or vaccine immunity
- Too many vaccines
- Boosters. Why?
- Is it even needed?

## Discussion

### Principal Findings

Our study aimed to develop and validate a message library for a social marketing campaign to increase COVID-19 vaccination among African Americans. The goal was to provide African American adults and parents with theory-based, culturally appropriate messaging on COVID-19 vaccines to motivate vaccine uptake. We described a multiphase process using community engagement approaches for the message library development with the HBM [31], the TRA [30], and Kreuter’s [32] cultural targeting strategies serving as the conceptual frameworks. Our existing library allowed us to expeditiously adapt the messaging to meet the needs of African Americans. This process can be used by researchers and health care professionals to inform the development of culturally appropriate messages.

Use of formative research to build theory-based, culturally appropriate messaging while applying community engagement principles is critical for communities to play an active role in disease prevention and control measures, such as COVID-19 vaccination [26,49]. This method holds great promise in addressing health disparities, yet is in its infancy [50,51]. Applying Boyer et al’s [52] multilevel approach to stakeholder engagement, we had community member involvement at all phases and varying levels to develop a message library to promote COVID-19 vaccination among African Americans. Engagement approaches included formation of a community-academic partnership, a CAP, and inclusion of community interviewees. Having the community-academic partnership and CAP allowed the community voice to be at the root of the messaging. Using each engagement approach, there was a balance of power to ensure that there was bidirectional communication and a deliberative process to foster respect, and even trust in some instances [53]. Furthermore, this process increased the likelihood of achieving cultural appropriateness of the messages.

Content validation has been recognized as a necessary component of message development and is highly valued [54]. The feedback provided by experts in the content review process was used to evolve the library with accurate and relevant messages. Furthermore, the suggestions for modification enriched the messages. Messages were further tested with a purposeful sample of African Americans for cultural appropriateness (ie, evidential, linguistics, peripheral, and sociocultural strategies). Our results indicated that African American adults and parents viewed the messages positively and indicated that the messages were *persuasive*, *useful*, and *trustworthy*. Feedback yielded distinct strategies to increase relevance, comprehension, and appeal. It is important to understand the target audiences’ response early to determine the likelihood of message effectiveness for the intervention [55].

Using this feedback from a multiphase process, our final message library yielded 18 message subsets for adults, and there were 17 message subsets for parents that were grounded in theory and cultural-targeting strategies. There were 3 preferred modes (ie, messages, images, and videos) for African American adults and parents. Studies demonstrate that multiple modes of communication are effective in increasing health literacy among populations, and plain-language messages, pictures, and videos are commonly cited, particularly in the context of community-level interventions [56]. We believe that this approach will be effective in reaching different characteristics of individuals.

### Strengths

A major strength of this study is the use of theory and culturally appropriate strategies inclusive of community engagement to develop the COVID-19 message library for African American adults and parents. We used different levels of community engagement (ie, community-academic partnership, CAP, and interviewees) to ensure that the messages met the needs of our target population. In addition, we equipped the community with information about COVID-19 vaccines to ease concerns postvaccination or to make an informed decision about getting the vaccine. Furthermore, these individuals can now serve as education resources to their communities.

The next step in our partnership will be to test these messages in a 5-month social marketing campaign in a pilot study. Specifically, these messages are used on a website to provide information on COVID-19 vaccines. Shortened versions of these messages are used to market the website. We will evaluate the impact on attitudes, willingness, and self-reported vaccination status to be reported in a future manuscript. If the intervention demonstrates effectiveness, it could prove that theory-based, culturally appropriate messages in a social marketing campaign can be used as a motivational tool among African Americans.

### Limitations

This study has limitations. Messages may not be generalizable to African Americans outside the southeastern United States. We had a small, purposeful sample, yet findings explained diverse perspectives to ensure messages encompassed multiple viewpoints toward the vaccine. There is potential for selection bias among content experts as they are medical professionals and clinicians from different disciplines and with clinical or research expertise. Furthermore, lack of access (ie, geographical barriers) to the vaccine could prevent uptake regardless of other concerns being addressed.

### Conclusion

Vaccine hesitancy continues to negatively impact COVID-19 vaccination among African Americans. Effective interventions are needed to increase vaccine uptake. We believe we have developed validated and pretested theory-based, culturally appropriate messages that can be motivational in different interventions aimed at increasing the COVID-19 vaccination rate among African Americans.

## Appendix

Multimedia Appendix 1 Methods and results of the literature search.

Multimedia Appendix 2 Qualitative quotations for themes.

## Abbreviations

CAP: community advisory panel
CHEN: Congregational Health and Education Network
EUA: Emergency Use Authorization
GBS: Guillain-Barré syndrome
HBM: Health Belief Model
mRNA: messenger RNA
SMOG: Simple Measure of Gobbledygook
TRA: Theory of Reasoned Action
