# Supplementary material for: Development of a Theory-Based, Culturally Appropriate Message Library for Use in Interventions to Promote COVID-19 Vaccination Among African Americans: Formative Research
Source: JMIR Form Res. 2022 Jul 28;6(7):e38781. doi: 10.2196/38781 (PMC9337618; doi:10.2196/38781)
Supplement: Multimedia Appendix 1 [file formative_v6i7e38781_app1.docx]

**Appendix File 1**

**Literature Review**

Narrative review of factors influencing COVID-vaccine hesitancy and informational needs to inform theory-based, culturally-appropriate messages for interventions to increase COVID-19 vaccination rates among African Americans.

**Methods**

A systematic literature search applying a purposive search was conducted in PubMed, CINAHL, PsycINFO and Web of Science databases up to from May 2020 to March 2022. This is not a traditional approach yet articles are selected based on specific concepts [1] Articles were identified using a combination of text to inform the COVID-19 message library for use in interventions for African Americans, Two concepts were the foci of the search. Concept 1 related to factors contributing to COVID vaccine hesitancy. The terms “vaccine”, “vaccination”, “COVID-19 vaccine”, “hesitancy”, “confidence”, “SARS-CoV-2”, “COVID”, “COVID-19”, “pandemic”, “African American”, “Black”, “black population”, and “Black American” were used in the search. Concept 2 related to communication around COVID-19 vaccination. The terms “African American”, “Black”, “Black population” “Black American”, “SARS-CoV-2”, “COVID-19”, “pandemic”, “COVID-19 vaccine”, “vaccination”, “communication”, “culturally-appropriate”, “message”, “messenger”, “theory”, and “framework” were used in the search. The search was completed when no new characteristics emerged around these two concepts.

**Results**

This search yielded 94 related to factors influencing COVID-19 vaccine hesitancy among African Americans and 12 informing current and future communication strategies. Articles were not considered if: 1) there was overlap; 2) redundancy in results, and 3) lack of relevancy. We selected a subset of 10 articles. A summary of the findings are provided below.

**Studies reporting factors associated with COVID-19 vaccine hesitancy among African Americans.** Among African American adolescents aged 15-17, In a qualitative study, Budhawani et al.[2] found that hesitancy was related to fear of side effects, institutional distrust, misinformation, and community leader and older family member influence among African American adolescents aged 15 to 17 years. Bogart et al.[12] conducted a cross-sectional survey in a national sample of African Americans and found that fear of harm and side effects related to the vaccine along with ones’ social network negatively influences vaccine confidence. Carson et al.[3] conducted a qualitative study among multi-ethnic communities and found that unethical history of research and speed of vaccine development as common concerns related to vaccine hesitancy. Similar, Momplaiser et al[4] qualitative findings described concerns with the accelerated timeline for vaccine development and limited data on short- and long-term side effects. In addition, participants cited mistrust in the medical establishment and the political environment promoting racial injustice as barriers.

Moore et al.[5] further found in a cross-sectional study that vaccine-resistant participants were more likely to have experienced housing insecurity due to COVID-19 and younger when compared to both hesitant and acceptant participants [5]. Majee et al.[9] found that the unethical historical research conducted by the government and pharmaceutical companies as factors associated with vaccine hesitancy. Other factors included uncertainty about vaccination (vaccines' safety, efficacy, and necessity), social media misinformation, and political affiliation. Kerrigan et al.[10] qualitative findings documented a lack of sufficient, accurate information about COVID-19 vaccines, inquiries about the science, mistreatment in healthcare among African American and African immigrant participants.

**Studies reporting information needs, current sources, and potential strategies to increase COVID-19 vaccine uptake among African Americans.** Carson et al. found that African Americans wanted data on safety and effectiveness of the vaccine among African Americans [3]. In Kerrigan et al.[10] qualitative study, African Americans message preferences included: 1) acknowledgement of past research abuses and differences in research practices today; 2) emphasis on returning to a normal; 3) convey “element of choice” and not being “forced”. Trusted information sources are key in delivering vaccine information to the African American community. Providers have been found as a trusted information source among African Americans across many studies [3, 6]. Woko, Seigel, and Hornik [7] found that Black Americans had significantly higher levels of trust in social media and main stream media compared to non-Black respondents. In a study among middle-aged to older African Americans [8], TV and social media were identified as the primary information sources exposing participants to COVID-19 related information. In a U.S. survey, Kricorian and Turner [11] found that many Black and Hispanic respondents reported that COVID-19 vaccine endorsements from same-race medical professionals would increase their willingness to receive it. In addition, receipt of information on experiences of vaccine study participants who are of their own race and ethnicity was identified as a motivator [11].

**Conclusions:**

Evidence regarding message concepts to be used in culturally-appropriate, theory-based interventions among African Americans are limited. None of the studies in the review identified culturally-appropriate, theory-based concepts to be used in interventions to increase COVID-19 vaccination among African Americans. Results of this literature review was used to iteratively develop the messages using a constituent-involving process. This resulted in a library of messages that may be more likely to resonate with the target audience.

**References**

1. Brunton, G., Thomas, J., O’Mara, A., Jamal, F., Oliver, S., & Kavanagh, J. (2017). Narratives of community engagement: a systematic review-derived conceptual framework for public health interventions. BMC Public Health, 17(1), 944.
2. Budhwani, H., Maycock, T., Murrell, W., & Simpson, T. (2021). COVID-19 vaccine sentiments among African American or black adolescents in rural Alabama. Journal of Adolescent Health, 69(6), 1041-1043.
3. Carson, S.L., Casillas, Y., Castellon-Lopez, L.N., et al., (2021). COVID-19 Vaccine Decision-making Factors in Racial and Ethnic Minority Communities in Los Angeles, California. JAMA Network Open, 4(9), e2127582-e2127582.
4. Momplaisir F, Haynes N, Nkwihoreze H, Nelson M, Werner RM, Jemmott J. Understanding drivers of coronavirus disease 2019 vaccine hesitancy among Blacks. Clin Infect Dis 2021 Nov 16;73(10):1784-1789.
5. Moore, J.X., Gilbert, K.L., Shah, S., et al. (2021). Correlates of COVID-19 Vaccine Hesitancy among a Community Sample of African Americans Living in the Southern United States. Vaccines, 9(8).
6. Sullivan, M.C., Mistler, C., Copenhaver, MM. et al. (2022). Race, trust, and COVID-19 vaccine hesitancy in people with opioid use disorder. Health Psychology, 41(2), 115-120.
7. Woko, C., Siegel, L., & Hornik, R. (2020). An Investigation of Low COVID-19 Vaccination Intentions among Black Americans: The Role of Behavioral Beliefs and Trust in COVID-19 Information Sources. J Health Commun, 25(10), 819-826.
8. Tang L, , York, FN, and Zou, W. (2021). Middle-Aged and Older African Americans’ Information Use during the COVID-19 Pandemic: An Interview Study. Front Public Health, 9, 709416.
9. Majee W, Anakwe A, Onyeaka K, Harvey IS. The past is so present: understanding COVID-19 vaccine hesitancy among African American adults using qualitative data. J Racial Ethn Health Disparities 2022 Feb 19:1-13.
10. Kerrigan D, Mantsios A, Karver TS, Davis W, Taggart T, Calabrese SK, et al. Context and considerations for the development of community-informed health communication messaging to support equitable uptake of COVID-19 vaccines among communities of color in Washington, DC. J Racial Ethn Health Disparities 2022 Feb 03:1-15.
11. Kricorian, K. & Turner, K. (2021). COVID-19 Vaccine Acceptance and Beliefs among Black and Hispanic Americans. PLoS One, 16(8), e025612
12. Bogart, L.M., Dong, L., & Gandhi, P. Klein DJ, Smith TL, Ryan S, et al. COVID-19 vaccine intentions and mistrust in a national sample of Black Americans. J Natl Med Assoc 2022 Jan;113(6):599-611
