# Supplementary material for: Development of a Theory-Based, Culturally Appropriate Message Library for Use in Interventions to Promote COVID-19 Vaccination Among African Americans: Formative Research
Source: JMIR Form Res. 2022 Jul 28;6(7):e38781. doi: 10.2196/38781 (PMC9337618; doi:10.2196/38781)
Supplement: Multimedia Appendix 2 [file formative_v6i7e38781_app2.docx]

**Appendix File 2**

**Qualitative Quotes for Themes in Phase V**

| **Table 2: Sample Quotations per Theme** | | |
| --- | --- | --- |
| **Quote Number** | **Concern** | **Quotation** |
| ***Theme 1: Community Overall Views on the Messaging*** | | |
| 1.01 | Natural Immunity | "Yeah. I feel like I've been very informed about just how effective of the vaccine is and like I said, I've never been 100% against it but just more hesitant, but this is really kind of moving me away from being so hesitant to really considering getting vaccinated in the near future.” (P18, Adult, Not vaccinated) |
| 1.02 | Vaccine Cause Serious Health Problems | "Wow, I've never heard about this information in the first bullet, over 30 years of research has led to the development of the COVID-19 vaccines, that's very interesting information. And the statistics about the vaccine saving over 279,000 lives and preventing 1.25 million hospitalizations, that's good information. The risk of an allergic reaction two to five people, that's good information. And I like the part where it talks about the side effects that you will have with the COVID-19... and this is really with any type of vaccinations, even with getting the flu vaccination.” (Participant 18, Adult, Not vaccinated) |
| 1.03 | Too many vaccines | "I think it's good. I think that the example of how many vaccines you get at that time is really an eye opener, and nothing happened to you then. You had to have how many before we got to take you home from the hospital? And it's the same concept, you were premedicated because you knew you were going to have a fever, you knew you were going to be sore, you knew you weren't going to rest good or eat good. It's the same, I think, concept now as when you were baby.” (Participant 11, Parent, Not vaccinated)" |
| ***Theme 2: Questions and Information Needs*** | | |
| 2.01 | Question needing the vaccine | "What about adding something in there about the reinfection? Because a lot of people have in their mind that because they... infection, because a lot of people have in their mind that because they've been infected, they don't need to be vaccinated. They're, I've got an antibody, that's what I hear people...I've already had it, I don't need a vaccine." (Participant 11, Parent, Not vaccinated)" |
| ***Theme 3: Suggestions for Additional Content*** | | |
| 3.01 | Vaccine Too New | "I think also given the information on how other vaccines have been developed or what instructions or how they are instructed to talk to the immune system. Because am I wrong? Is this the first time that the mRNA has been used? The messenger RNA?” (Participant 20, Adult, Not vaccinated)" |
| 3.02 | NA | "No, I think that that language is definitely sticky, and people wonder what are you going to tell me? But, when you come at people with just facts, it's like, They're not talking to me, I've already made up my mind. But, if you pose an open-ended question, did you know that COVID-19 may cause erectile dysfunction? How many any guys are going to look at that and think, Johnsons in trouble? That may be a catch point for another demographic of people at risk, or a demographic of people that have already tried to say that they don't want the vaccine. If they were to be engaged with a bullet point that's not been so much in the forefront like that. That's something that just totally... It still sticks with me, or did you know that... A question and answer, the top five myths of or associated with COVID-19 vaccinations. You have those listed there, that engages people to... It's quick, it's easy, and it engages them to learn, or want to know more about it.” (Participant 11, Parent, Not vaccinated) |
| 3.03 | Vaccine Too New | "Now you got this jab out here that you're not fully ... The powers that be are not fully disclosing in my opinion, are not giving us all the facts or all the details, but yet they want us to rush and get it. I need full disclosure. I don't need no misinformation, half information, information held back, whatever. I need to know everything if you want me to put this into my body. Because if it's something that can do me more harm than good in the long run, I need to know that.” (Participant 20, Adult, Not vaccinated) |
| 3.04 | Question Vaccine Effectiveness | "...a comparison to show what an unvaccinated versus a vaccinated person went through with the vaccine or without the vaccine. That to me would be a bigger statement than statistics. So maybe a visual rather than a reader, because that doesn't really hit home like a picture of somebody that's sick maybe at home versus somebody that's on day 15 on a ventilator and an ECMO.” (Participant 11, Parent, Not vaccinated) |
| 3.05 | Vaccine Safety Concerns | "And what happened in the cases? I guess you can mention that. Was it with the drug company just saying, Oops, sorry? Or just some... I don't know.” (Participant 2, Adult, Not vaccinated) |
| 3.06 | NA | “...One thing that I've learned with COVID is to build your immune system and that's what I have been doing, is building my immune system. I've been taking multi gummies for two years and so I started taking it even before COVID hit. But one of the things that I learned is when you have viruses like this, it's good to keep your immune system built up.” (Participant 18, Adult, Not vaccinated) |
| 3.07 | NA | "I know with people that are vaccinated, there are breakthrough cases and I know a lot of times with that that's people who are more at high risk. But me in my situation, I don't feel like that there would be... I think even if I caught COVID, I would be what you would consider asymptomatic, have mild symptoms. I don't get sick a lot anyway because like I said, I keep my immune system up and now if I had a lot of health issues, then I would probably be a little bit more open to getting the vaccine.” (Participant 18, Adult, Not vaccinated) |
| 3.08 | Vaccine Cause Serious Health Problems | “...It's just like, I don't take the flu vaccine every year. I mean, I just felt like I have an immune system that if I caught COVID, I would be fine. I'm a runner, you know what I mean? I'm a healthy individual. Even if COVID was a every year shot, I don't think it would be something that I would consider because I don't take the flu shot. And I'm not anti-vaccine. Like I said, I'm not anti-vaccine I have the pneumonia vaccine, I have HPV vaccine. I have vaccines. I've been vaccinated for a lot of stuff, but it is just the stuff that I feel like will kill me is what I'll take a vaccine for.” (Participant 19, Adult, Not vaccinated) |
| ***Theme 4: Suggestions to Increase Comprehension, Relevance, and Trustworthiness*** | | |
| 4.01 | Question Vaccine Effectiveness | “...So when you say high risk, what does that mean for you? Because everybody wants to be sick because everybody thinks they need it. So I think some examples of what high risk is, would be good, to clear up some of that confusion or frustration or anxiety.” (Participant 11, Parent, Not vaccinated) |
| 4.02 | NA | "...You could have testimonials. People who've been infected. People who haven't been infected. People who've been vaccinated. People who had complications with vaccines. All of those people need to be included so that educated decisions can be made for people in every situation.” (Participant 11, Parent, Not vaccinated) |
| 4.03 | NA | "I feel like testimonial would be, it's kind of farfetched. I feel like it would be too stressed in that sense, having testimonial. I've just been having the thoughts in my head that they're just made up or fake or whatever. I'm basing my belief on fact. Fact and research definitely could help and go a long way.” (Participant 25, Parent, Not vaccinated) |
| 4.04 | NA | "Yeah. I think probably them working together or something, because personally I do believe my doctor 100%. I do not doubt his judgment. So perhaps, I don't know, let them in on the whole process. I don't know how that would work exactly. But perhaps if my doctor came to me and was like, Oh, I actually do know about this stuff firsthand. And convincing me or people involved in the process. So I'd be like, Oh I do trust this person's judgment, so if they do say, This is how this stuff works or this is how it was made, and it's healthy for me, then, yeah. Yeah. I'll definitely trust. Yeah, I'll definitely trust the process.” (Participant 24, Parent, Not vaccinated) |
